# 3D models of glioblastoma interaction with cortical cells

**DOI:** 10.3389/fbioe.2023.1150772

**Published:** 2023-03-09

**Authors:** Md Joynal Abedin, Sharon K. Michelhaugh, Sandeep Mittal, Yevgeny Berdichevsky

**Affiliations:** ^1^ Department of Bioengineering, Lehigh University, Bethlehem, PA, United States; ^2^ Fralin Biomedical Research Institute, Virginia Tech, Roanoke, VA, United States; ^3^ Department of Electrical and Computer Engineering, Bethlehem, PA, United States

**Keywords:** 3D glioblastoma modeling, host cell-tumor cell interaction, glioblastoma, tumor- associated epilepsy (TAE), calcium imaging, cortical neuron, neuronal activity, matrix-free

## Abstract

**Introduction:** Glioblastoma (GBM) invasiveness and ability to infiltrate deep into the brain tissue is a major reason for the poor patient prognosis for this type of brain cancer. Behavior of glioblastoma cells, including their motility, and expression of invasion-promoting genes such as matrix metalloprotease-2 (MMP2), are strongly influenced by normal cells found in the brain parenchyma. Cells such as neurons may also be influenced by the tumor, as many glioblastoma patients develop epilepsy. *In vitro* models of glioblastoma invasiveness are used to supplement animal models in a search for better treatments, and need to combine capability for high-throughput experiments with capturing bidirectional interactions between GBM and brain cells.

**Methods:** In this work, two 3D *in vitro* models of GBM-cortical interactions were investigated. A matrix-free model was created by co-culturing GBM and cortical spheroids, and a matrix-based model was created by embedding cortical cells and a GBM spheroid in Matrigel.

**Results:** Rapid GBM invasion occurred in the matrix-based model, and was enhanced by the presence of cortical cells. Little invasion occurred in the matrix-free model. In both types of models, presence of GBM cells resulted in a significant increase in paroxysmal neuronal activity.

**Discussion:** Matrix-based model may be better suited for studying GBM invasion in an environment that includes cortical cells, while matrix-free model may be useful in investigation of tumor-associated epilepsy.

## 1 Introduction

Gliomas are a group of primary brain cancers that have poor patient prognoses due to lack of effective treatment. Glioblastoma (GBM), which is the most common and aggressive form of glioma, has a patient survival rate of less than 20% after 2 years (Ostrom et al., 2018). Surgical resection is used to remove the glioma tumor mass; however, due to glioma’s invasiveness and ability to infiltrate deep into the brain tissue, resection leaves some cancer cells causing tumor recurrence (Young et al., 2015). Chemotherapy and radiation are used after surgical resection in an attempt to destroy the leftover glioma cells, but the efficacy is unfortunately low (Johnson and O’Neill, 2012).

The ability of glioma to effectively invade the brain parenchyma beyond the border of the tumor mass has been a subject of significant research interest (Cuddapah et al., 2014; Alfonso et al., 2017; Vollmann-Zwerenz et al., 2020). A number of animal and *in vitro* models have been developed to investigate the mechanisms of glioma proliferation and invasion, and to test novel therapeutics (Lenting et al., 2017). While animal models come perhaps the closest to emulating processes occurring in the patients’ brain, they have a number of disadvantages, the chief of which are the difficulty of experimental access and relatively low throughput. To supplement the animal models, far simpler and more accessible *in vitro* models are used (Vollmann-Zwerenz et al., 2020). The design of an *in vitro* model must balance its simplicity and throughput with the model’s ability to replicate the tumor microenvironment in the brain (Wolf et al., 2019). Glioma invasion has been found to depend heavily on its microenvironment, occurring most rapidly along white matter tracts and blood vessels, although invasion through the gray matter can occur as well (Cuddapah et al., 2014). Behavior of glioma cells, including their motility, and expression of invasion-promoting genes such as matrix metalloprotease-2 (MMP2), has been found to be strongly influenced by normal cells found in the brain parenchyma: endothelial cells, microglia, astrocytes, and even neurons (Venkatesh et al., 2015; Hambardzumyan et al., 2016; Brandao et al., 2019). These stromal cells themselves can be influenced by the presence of glioma. In glioblastoma (GBM), 55%–65% of patients develop epilepsy (Englot et al., 2016). This may occur due to release of glutamate, which functions as an excitatory neurotransmitter, from glioma, which causes hyperexcitability in nearby neurons. In addition to cell-cell interactions, the composition and mechanical properties of the extracellular matrix (ECM) also exert a significant influence on glioma cells (Wolf et al., 2019).

The model that captures these interactions most accurately *in vitro* is perhaps the slice culture model. In this model, glioma cells, spheroids, or organoids are placed in a slice of the brain region and allowed to infiltrate over several days (Eyüpoglu et al., 2005; Aaberg-Jessenet al., 2018; Eisemann et al., 2018). Organotypic culture techniques preserve the cytoarchitecture of the slice, and thus the glioma cells are presented with an *in vitro* microenvironment for invasion that is close to that found in the brain. Organotypic slice cultures have an important disadvantage, however, in terms of the low numbers of slices that can be generated per dissected animal, and specialized culture techniques that are required. This model is therefore not suitable for high-throughput drug discovery. At the other end of the complexity spectrum is the widely used model of invasion where a glioma spheroid is placed into a three-dimensional (3D) hydrogel such as Matrigel (Vinci et al., 2015). Glioma cells rapidly invade the hydrogel in a manner that is easily imaged and quantified, and very large numbers of parallel experiments are possible. However, the realism of glioma-microenvironment interactions is sacrificed, as hydrogel does not contain any of the stromal cells that strongly interact with glioma cells (Herrera-Perez et al., 2018). More recently developed 3D models used co-cultures of glioma cells with at least one other cell type in an engineered microenvironment. Co-cultures of glioblastoma cells and endothelial cells in 3D engineered capillaries were used to model permeability of blood brain barrier (Marino et al., 2018). 3D models of tumor microenvironment were created by populating engineered 3D scaffolds with glioma or glioblastoma cells (Barin et al., 2022) and by bioprinting perfusable 3D co-cultures of glioblastoma cells with astrocytes, microglia, pericytes, and endothelial cells (Neufeld et al., 2021). Tumor cells exhibited different behavior, including response to treatment (Akolawala et al., 2022), in 3D environments of these models compared to 2D culture. However, data on the interactions between stromal cells such as neurons and glioma in engineered 3D environments remains limited.

In this work, we set out to create a high-throughput—capable model of glioblastoma proliferation and invasion in a 3D environment that enabled interactions between GBM and stromal cells. We used glioblastoma spheroids as a tumor model, similar to organotypic and Matrigel-based models described above. To create a 3D environment for glioblastoma invasion, we utilized two approaches: 1) matrix-free 3D aggregates of primary cortical cells (Ming et al., 2020), (Hasan and Berdichevsky, 2021), and 2) Matrigel-based 3D cultures of primary cortical cells. Both approaches benefit from higher experimental throughput compared to organotypic slice cultures. To the best of our knowledge, these approaches have not been previously used to create 3D co-cultures of cortical and GBM cells. We examined whether the 3D environments we created influenced glioblastoma cell behavior. We also investigated whether the presence of glioblastoma spheroid impacted the behavior of cortical cells. Our work illustrates a novel approach to concurrently model GBM invasion and neuronal hyperexcitability.

## 2 Materials and methods

### 2.1 Cell culture

*Rat primary cell culture preparation* Brains of postnatal day 0-1 Sprague–Dawley rat pups (Charles River Laboratories) were dissected in ice-cold Hanks’ balanced salt solution (HBSS, Gibco). Cortices were cut into fine pieces and digested in papain solution (Worthington Biochemical Corp.) at room temperature. Cortices were then triturated slowly in culture medium, and centrifuged in 30% Percoll (Sigma-Aldrich). The pellet was resuspended in cell culture solution [Neurobasal-A, 0.5 mM GlutaMAX, 30 μg/mL gentamicin, supplemented with B27, (Gibco)]. Cultures were incubated at 37°C and 5% CO_2_ after plating on substrates described below.

*Patient-derived GBM cell lines* Generation of GBM cell lines from patient tumors was described previously (Karki et al., 2020). Both 15-037 and 14-104s cell lines were isolated from newly-diagnosed patient tumors that were untreated at the time of collection. Both are IDH1 wildtype. 14-104s has a methylated MGMT promoter and is p53 wild type. 15-037 has an unmethylated MGMT promoter and has mutated p53.

### 2.2 Co-culture of cortical cells and GBM spheroids without Matrigel

**FIGURE 1:**
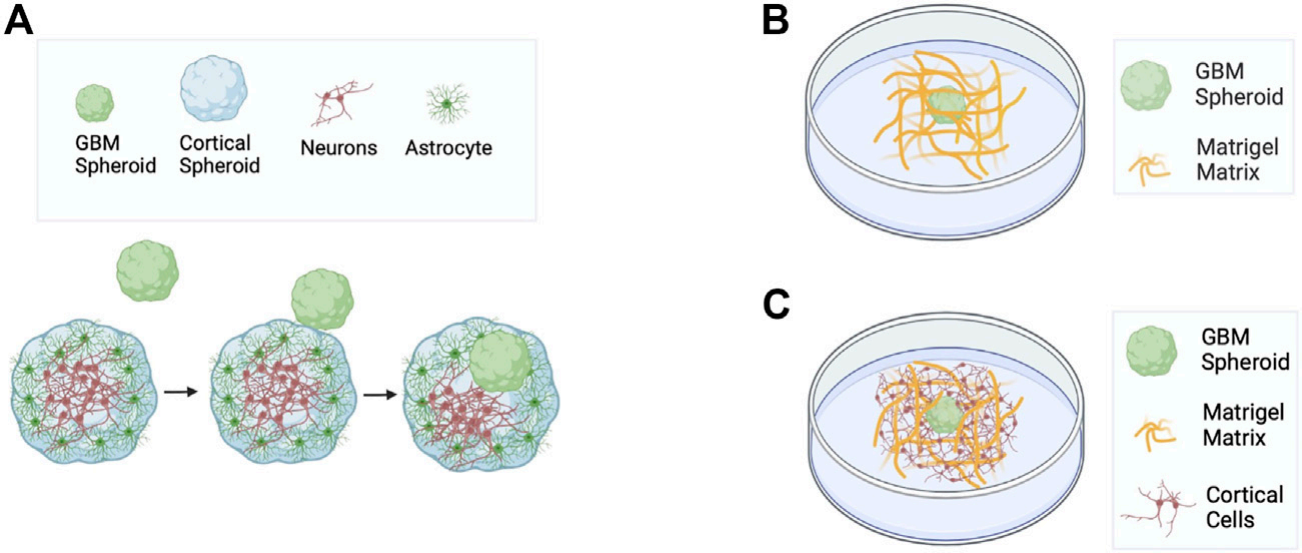
Schematics of GBM models used in this work. **(A)** Matrix-free co-culture of a cortical spheroid and a GBM spheroid. **(B)** GBM spheroid embedded in Matrigel matrix. **(C)** Co-culture of cortical cells and a GBM spheroid embedded in Matrigel matrix.

To prepare spheroids, 500 μL of molten agarose was pipetted into commercially available micro molds (12-81, Microtissues, Inc.) to make micro 3D petri dishes. Micro 3D petri dishes were placed in a 12-well plate (Corning), and plated with 400,000 primary cortical cells. After 2 days of incubation, dissociated primary cortical cells formed spheroids. GBM cells were cultured on a T75 flask with completed media composed of DMEM/F12 (Corning) supplemented with non-essential amino acids, 10% fetal bovine serum (Gibco), 0.5 mM GlutaMAX, and 30 μg/mL gentamicin. GBM cells were detached using Accutase (Innovative cell technologies, AM-105), and GBM spheroids were created by plating 10,000 GBM cells in an agarose-made micro 3D Petri dish. After GBM cells formed spheroids (typically in 2–3 days), GBM spheroids were transferred to dishes containing cortical spheroids (1 GBM spheroid and 1 cortical spheroid per well). GBM spheroid and cortical spheroid fused after 2 days of co-culture (Figure 1A). Time-lapse imaging of fusion of GBM spheroid and cortical spheroid was performed with Olympus IX 73 microscope. Co-cultures were imaged with an inverted phase contrast microscope (CKX 41, Olympus) on different DIVs using a 4x objective. Spheroid size was measured using ImageJ (NIH).

### 2.3 GBM spheroids in Matrigel

Polydimethylsiloxane (PDMS) base (Fisher Scientific, Cat# NC9285739) and curing agent were mixed at a 10:1 ratio. PDMS was then spin-coated on a silicon wafer to make a 200 µm thick PDMS film. Well with dimensions of ∼1.5 mm × 2.5 mm was cut into each PDMS film. PDMS wells were attached onto glass bottom 35 mm petri dishes coated with poly-D-lysin (PDL) (Sigma-Aldrich). GBM spheroids were created on agarose-made micro 3D petri dish as described above. 4 day old GBM spheroids were collected and mixed with ice-cold Matrigel (Corning, Cat# 354234) in a 200 µL tube on ice. Then 8 µL of Matrigel with GBM spheroids was plated into each PDMS well (Figure 1B). Culture was maintained in Neurobasal-A supplemented with B27, 0.5 mM GlutaMAX, and 30 μg/mL gentamicin (Gibco). GBM spheroids were imaged every 24 h. Some spheroids were imaged every 30 min for 72 h to determine the time course of invasion in the Matrigel matrix. The invaded area was calculated as the total area with protrusions minus the contour without protrusions using ImageJ (NIH) and plotted at different time points.

### 2.4 GBM spheroids in Matrigel with cortical cells

Rat primary cortical cells and 4 day old GBM spheroids were prepared as described before. They were then mixed with Matrigel (Corning, Cat# 354234) on ice at 10 million cortical cells per mL. 8 μL of this mixture (rat primary cells + GBM spheroids + Matrigel matrix) was plated into each PDMS well (Figure 1C). For each experiment, GBM spheroids in Matrigel without cortical cells were also plated in PDMS wells to serve as controls. Both types of cultures were maintained in Neurobasal-A/B27 media supplemented with 0.5 mM GlutaMAX and 30 μg/mL gentamicin. Cultures were imaged every 24 h until DIV 4 to evaluate effect of cortical cells on GBM invasion. Invaded area (total area—contour area) was plotted at different time points. Spheroids that merged/touched other spheroids were excluded from analysis.

For analysis of cortical cells’ movement relative to GBM spheroids, imaging was performed with Nikon BioStation IM until 96 h. Coordinates of the spheroid’s invasion mantle and cells’ position with time were exported from ImageJ to MATLAB to process the data further.

### 2.5 Viral infection

Rat primary cortical neurons were transfected with jRGECO1a, which is a genetically encoded calcium indicator using virus pAAV.Syn.NES-jRGECO1a.WPRE.SV40 [pAAV.Syn.NES-jRGECO1a.WPRE.SV40 was a gift from Douglas Kim & GENIE Project (Addgene plasmid # 100854; http://n2t.net/addgene:100854; RRID:Addgene_100854)] at titer 1.9 × 10^13^ GC/mL. GBM cells were tagged with an enhanced green fluorescent protein (eGFP) using virus pAAV-CAG-GFP [pAAV-CAG-GFP was a gift from Edward Boyden (Addgene plasmid # 37825; http://n2t.net/addgene:37825; RRID:Addgene_37825)] at titer 1.4 × 10^13^ GC/mL. Viral infection was performed on DIV 0.

### 2.6 Immunohistochemistry and imaging

Samples were fixed using 4% paraformaldehyde (PFA, Electron Microscopy Science) for 1 h and permeabilized in 0.3% triton X-100 (Sigma-Aldrich) on a shaker for 30 min. After permeabilization, samples were incubated in 10% goat serum (Gibco) in 0.05% triton X-100 in PBS blocking buffer for 1 h on a shaker. Primary antibodies were applied at 4°C on an orbital shaker at 55 RPM for 72 h. Secondary antibodies were applied for another 72 h. Co-cultures of cortical spheroids and GBM spheroids were fixed on DIV 13, stained using primary antibodies to Anti-NeuN, clone A60, Alexa Fluor 488 conjugated (1:200 dilution, Millipore Sigma, Cat# MAB 377X) and Anti-GFP (chicken antibodies, IgY) (1:500 dilution, Aves, Cat# GFP-1010) followed by secondary antibody Goat anti-Chicken IgY (Aves, Cat# F-1005). Co-cultures embedded in Matrigel matrix were fixed on DIV 7 and stained using purified anti-neurofilament marker SMI-312 antibody (1:500 dilution, Biolegend, Cat# 837904) and anti-MMP2 antibody (1:500 dilution, Abcam, Cat# ab92536). Alexa fluor 488 (Invitrogen, Cat# A21121) and Alexa fluor 568 (Life Technologies, Cat# A11011) were used as secondary antibodies respectively. All secondary antibodies were used at 1:200 dilution. Nuclei were labeled with DAPI (Invitrogen, Cat# R37606).

Stained cultures were sandwiched between two coverslips with Fluoro-gel (Electron microscopy sciences, Cat# 17985-10) and imaged using a confocal microscope (Zeiss LSM 510 META, Germany) with a 20x objective at constant exposure and laser power. The distance between optical slices was 3 µm and samples were imaged over their entire depth.

Stained matrix-free co-cultures were transferred to a PDMS well of a thickness of 200 µm before they were sandwiched between two coverslips. Co-cultures of spheroids were imaged using a confocal microscope with 2 µm optical slices.

### 2.7 Neuronal activity imaging and analysis

Cultures expressing jRGECO1a were taken to a humidified recording chamber maintained at 37°C with 5% CO_2_ on a microscope (IX73, Olympus) to record the calcium activity of neurons. 10x and 4x objectives were used for neuronal activity imaging for co-culture with and without Matrigel matrix, respectively. Videos were recorded at 5 frames per second with 640 × 360 (after 3 × 3 binning) resolution and 8 bits per pixel with sCMOS camera (Thorlabs). Regions of interest (ROIs) were drawn to include the spheroid for co-culture without Matrigel, and for co-culture with Matrigel, ROIs were drawn to include individual cells. Video files were converted to raw mean grey values using ImageJ. Baseline fluorescence *F*_*0*_ was calculated in MATLAB using the Asymmetric least square mean smoothing method (Eilers and Boelens, 2005). This method allowed us to find a variable baseline F_0_ without any prior knowledge of the peak regions of the signal. If F is the instantaneous fluorescence, fluorescent change over baseline was calculated by

$$\frac{\Delta F}{F}=\frac{\left(F-Fo\right)}{Fo}$$ (1)

The threshold was set to exclude optical noise, and was same for all activity analyses. Cumulative activity duration and cumulative area under the curve above the threshold were obtained.

### 2.8 Statistics

Two-tailed paired *t*-test was used to measure the statistical significance in the analysis of cortical cells’ motion relative to the GBM invasion mantle. Two-tailed Kolmogorov–Smirnov (KS) test was performed in all other analyses. A *p*-value < 0.05 is considered significant.

## 3 Results

### 3.1 Matrix-free cortical-GBM co-cultures

**FIGURE 2:**
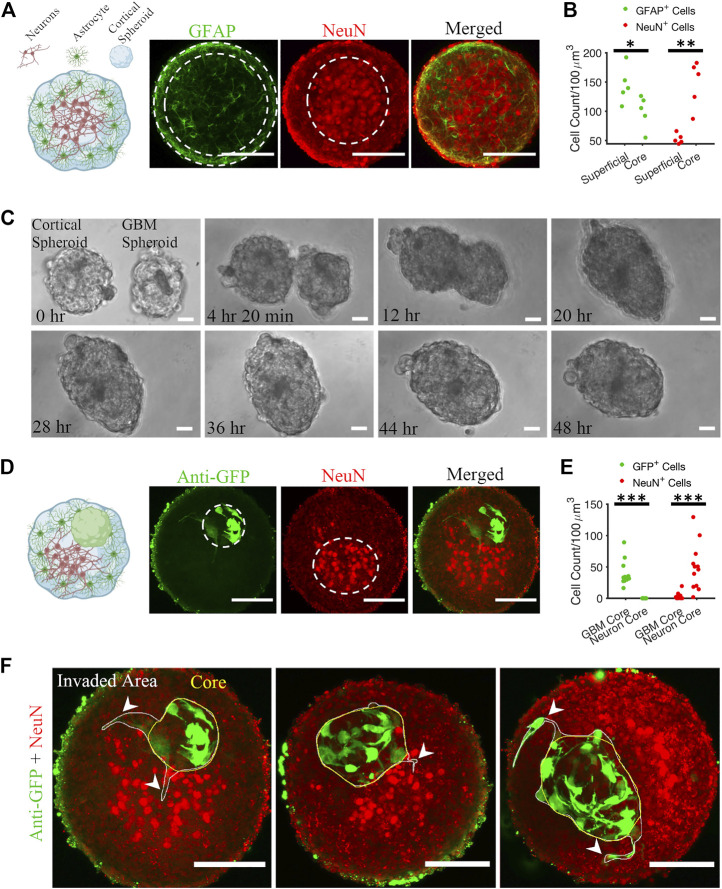
Matrix-free co-culture of a cortical spheroid and GBM spheroid. **(A)** Left: schematic of the internal morphology of a cortical spheroid. Right: representative z-projection of anti-GFAP and anti-NeuN stained fluorescent images shows a neuronal core in the center and astrocytes in the superficial layer. **(B)** Comparison of GFAP^+^ and NeuN^+^ cell count in core and superficial layer, *n* = 5 spheroids. **(C)** Time-lapse imaging of merging GBM and cortical spheroids. **(D)** Left: schematic of the internal morphology of a merged cortical-GBM spheroid. Right: representative fluorescent images of cortical-GBM spheroid stained with anti-GFP (GBM cells expressed GFP) and anti-NeuN antibodies. The cluster of GBM cells pushed the neuronal core to an off-center position. **(E)** Comparison of GFP^+^ and NeuN^+^ cell count in core and superficial layer, *n* = 12 spheroids. **(F)** Three examples of invasion of GBM spheroids in matrix-free co-culture (merged cortical-GBM spheroid). Arrowheads show invasive protrusions. Scale bar = 100 μm in all panels. Two-sample Kolmogorov–Smirnov (KS) test was used to measure statistical significance. *p < 0.05, **p < 0.01 and ***p < 0.001.

First, we plated 400,000 cortical cells into an agarose micro 3D petri dish. After 2 days of incubation, cortical cells formed spheroids with cross-section area of 30,685 ± 10,782 μm^2^ (mean ± standard deviation, *n* = 81). We fixed the cortical spheroids on DIV 10. Astrocytes and neurons self-organized inside a spheroid such that neurons formed a core at the center of the spheroid and astrocytes were predominantly found in the superficial layer (Figures 2A, B). Z-projection of 30 μm stacks using maximum intensity was used to draw the region of interest (ROI) of the NeuN core, and GFAP superficial layer, and respective cells were counted. The number of GFAP-positive (GFAP^+^) cells in the superficial layer was significantly higher than the number of GFAP^+^ cells in the core (KS test, *p* < 0.05). In contrast, the number of NeuN-positive (NeuN^+^) cells was significantly higher in the core than in the superficial layer (KS test, *p* < 0.01).

Second, 10,000 patient-derived GBM cells were plated into an agarose micro 3D petri dish on the day before cortical cells plating. 3 days after plating GBM cells, resulting GBM spheroids with cross-section area of 25,694 ± 10,843 μm^2^ (*n* = 78) were transferred to cortical spheroids at 1-to-1 ratio per well. Cortical and GBM spheroids merged within 48 h (Figure 2C; Supplementary Video S1). We fixed the cortical-GBM co-cultures on DIV 13. Confocal imaging of the co-culture showed that GBM cells (expressing GFP and stained with an anti-GFP antibody) were inside the cortical spheroid and near the neurons, but not mixed with them (Figure 2D). Nuclei of GFP-positive GBM cells and nuclei of NeuN-positive neurons formed cores (clusters) in different positions (Figure 2D). Z-projection of 30 μm stacks using maximum intensity was used to draw the region of interest (ROI) of the NeuN core and GBM core. We counted the GFP^+^ and NeuN^+^ cells at the GBM core and neuron core. There were no GFP^+^ cells at the neuron core (Figure 2E). The number of NeuN^+^ cells in the neuron core was significantly higher than the number of NeuN^+^ cells in the GBM core (KS test, *p* < 0.001) (Figure 2E). Culture of merged GBM and cortical spheroids exhibited little GBM invasion into either neuron core or superficial layer (Figure 2F). However, some GBM cells showed invasive protrusion (Figure 2F). This may be because GBM cells invade primarily in the presence of ECM (Vinci et al., 2015).

### 3.2 GBM cells facilitate the activity of cortical neurons in matrix-free co-cultures

**FIGURE 3:**
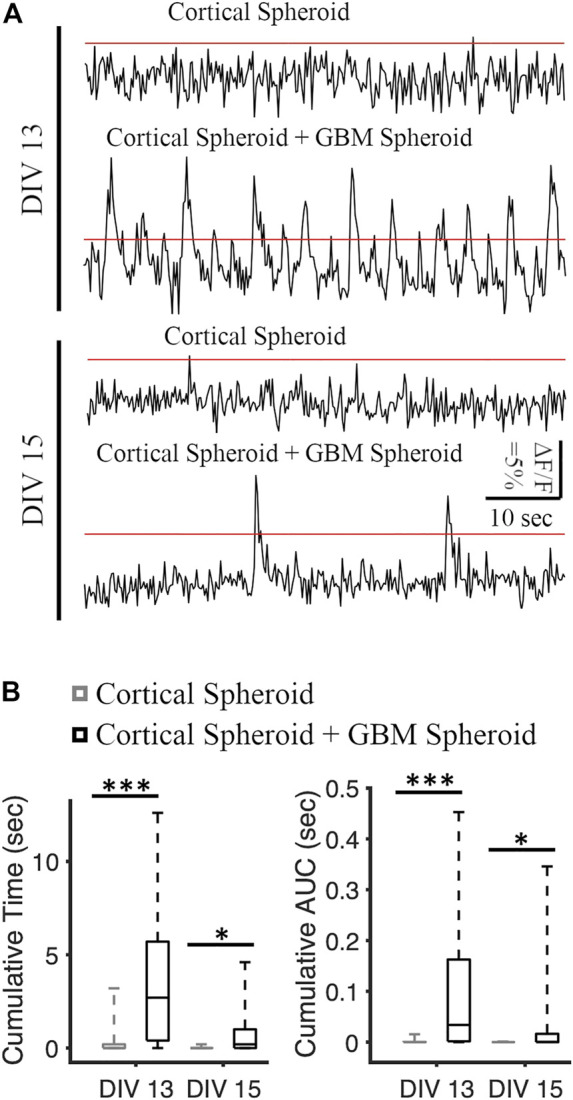
Analysis of spontaneous neuronal activity in cortical and cortical-GBM spheroids. **(A)** Representative traces of jRGECO1a (red fluorescent genetically encoded calcium indicator) in cortical spheroid and cortical spheroid + GBM spheroid in DIV 13 and DIV 15. The red lines represent the threshold of 5% ΔF/F. Scale bar: 10 s, 5% ΔF/F. **(B)** Boxplots of cumulative time and area under the curve (AUC) above threshold on different DIVs. N = 26 cortical spheroids and 24 cortical-GBM spheroids in DIV 13. N = 24 cortical spheroids and 22 cortical-GBM spheroids. KS test was used to measure statistical significance. *p < 0.05 and ***p < 10^–5^.

We recorded the neuronal activity of cortical spheroids and merged cortical-GBM spheroids by measuring fluorescence changes in neuronally-expressed jRGECO1a (Figures 3A, B). On DIV 13, the cumulative activity duration of neurons in cortical-GBM was significantly higher than the cumulative activity duration of neurons in cortical spheroids (2.7 [0.4, 5.7] seconds and 0 [0 0.2] seconds, respectively, median [Q1 (1st Quartile), Q3 (3rd Quartile)], KS test *p* < 0.001) (Figure 3B). On DIV 15, the cumulative activity duration was significantly higher in cortical-GBM spheroids than in cortical spheroids (0.2 [0, 1] sec and 0 [0 0] sec, respectively, KS test *p* < 0.05) (Figure 3B). On DIV 13, the cumulative area under the curve above 5% ΔF/F was significantly higher for cortical-GBM spheroids compared to cortical spheroids (0.034 [0.002, 0.163] sec and 0 [0 0] sec, respectively, KS test *p* < 0.001) (Figure 3B). On DIV 15, the cumulative area under the curve above 5% ΔF/F was significantly higher in cortical-GBM spheroids compared to cortical spheroids (0.001 [0, 0.016] sec and 0 [0 0] sec, respectively, KS test *p* < 0.05) (Figure 3B). This increase in the neuronal activity in cortical-GBM spheroids indicates the presence of interactions between neurons and GBM cells that may contribute to tumor-causing epilepsy (Englot et al., 2016).

### 3.3 GBM spheroids in Matrigel

**FIGURE 4:**
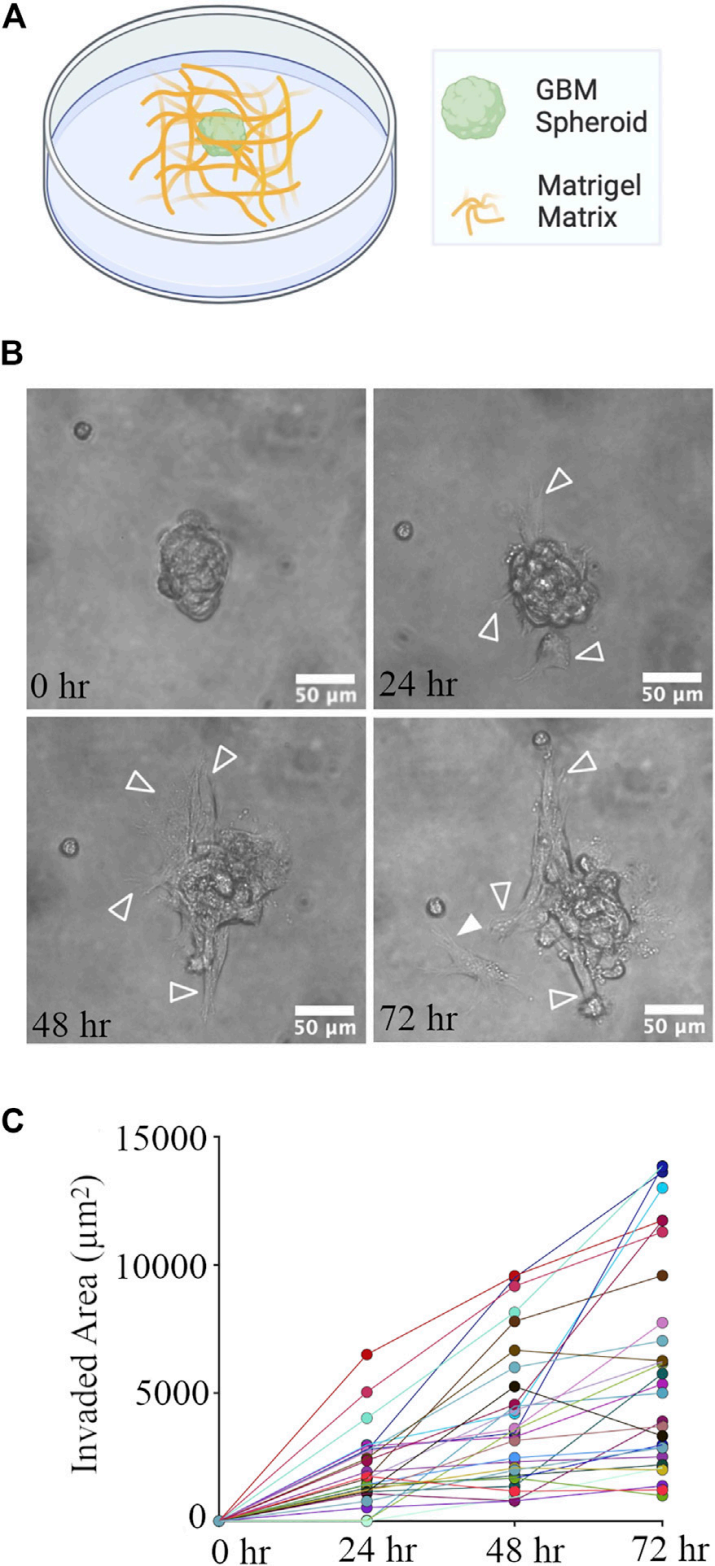
GBM Spheroid embedded in Matrigel matrix. **(A)** Schematic of a GBM spheroid in Matrigel. **(B)** Time-lapse imaging of a GBM spheroid. Arrowheads show invasive protrusions, and the solid arrowhead shows a detached GBM cell. Scale bar is 50 μm. **(C)** Quantification of the invaded area at different time points. N = 28 GBM spheroids at each time point.

GBM spheroids were created using patient-derived glioma cells. 4 days old GBM spheroids (cross-section area: 4,819 ± 1786 μm^2^, *n* = 44) were embedded in the Matrigel matrix (Figure 4A). Phase contrast images of GBM spheroids show extended protrusions after 24 h (Figure 4B). At the 72-h time point, GBM cells detached from the spheroid (Figure 4B). Invasion (extension of protrusions) by cells in GBM spheroids progressed during the first 72 h (Figure 4C). Correlation between the initial size of the spheroids and invaded area was weak (Pearson correlation *r* ^2^ = 0.143, *p* = 0.04); therefore, we used absolute measurement of the invaded area in this and the following experiments. We then proceeded to evaluate the effect of cortical cells on GBM invasion in Matrigel.

### 3.4 GBM spheroids and cortical cells in Matrigel

**FIGURE 5:**
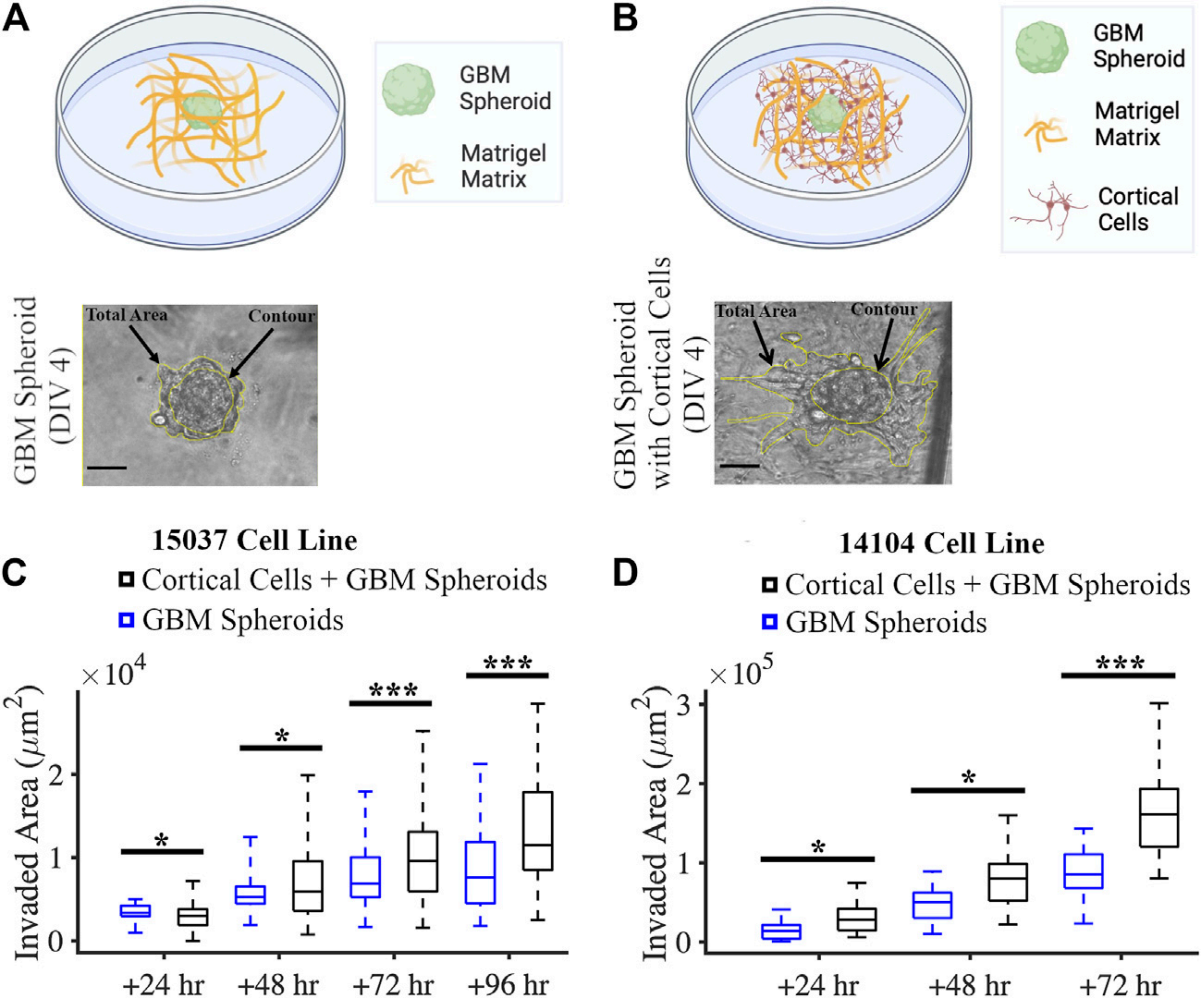
Co-culture of GBM spheroid embedded in Matrigel matrix mixed with cortical cells. **(A)** Schematic and a phase micrograph of a GBM spheroid in Matrigel on DIV 4. **(B)** Schematic and a phase micrograph of a GBM spheroid co-cultured with cortical cells embedded in Matrigel matrix on DIV 4. Phase micrographs illustrate the invaded area in each culture condition calculated as the difference between the total area and contour. **(C)** Boxplots of the invaded area of GBM spheroids with and without cortical cells over different time points for the 15-037 GBM cell line. N = 58 GBM spheroids without cortical cells and *n* = 74 GBM spheroids with cortical cells. KS test was used to measure statistical significance. *p < 0.05, **p < 0.01 and ***p < 0.001. **(D)** Boxplot of the invaded area of GBM spheroids with and without cortical cells over different time points for the 14-104s GBM cell line. N = 25, 19, 14 GBM spheroids without cortical cells at 24 h, 48 h, and 72 h, respectively. N = 18, 18, 14 GBM spheroids with cortical cells at 24 h, 48 h, and 72 h, respectively. KS test was used to measure statistical significance. *p < 0.05, **p < 0.01 and ***p < 0.001.

We created a co-culture system by mixing GBM spheroids and primary rat cortical cells with a Matrigel matrix (Figure 5B). We also plated GBM spheroids without cortical cells in Matrigel matrix in this experiment as control (Figure 5A). We evaluated the effect of cortical cells on the invasion of GBM spheroids using two GBM cell lines. For the 15-037 cell line, the invaded area of GBM spheroids in the presence of cortical cells was significantly higher than the invaded area of GBM spheroids without cortical cells at all time points except at 24 h (KS test) (Figure 5C). For the 14-104s cell line, invaded area was significantly higher for GBM spheroids in the presence of cortical cells compared to GBM spheroids only at all time points (KS test) (Figure 5D).

### 3.5 MMP expression changes due to cortical cells

**FIGURE 6:**
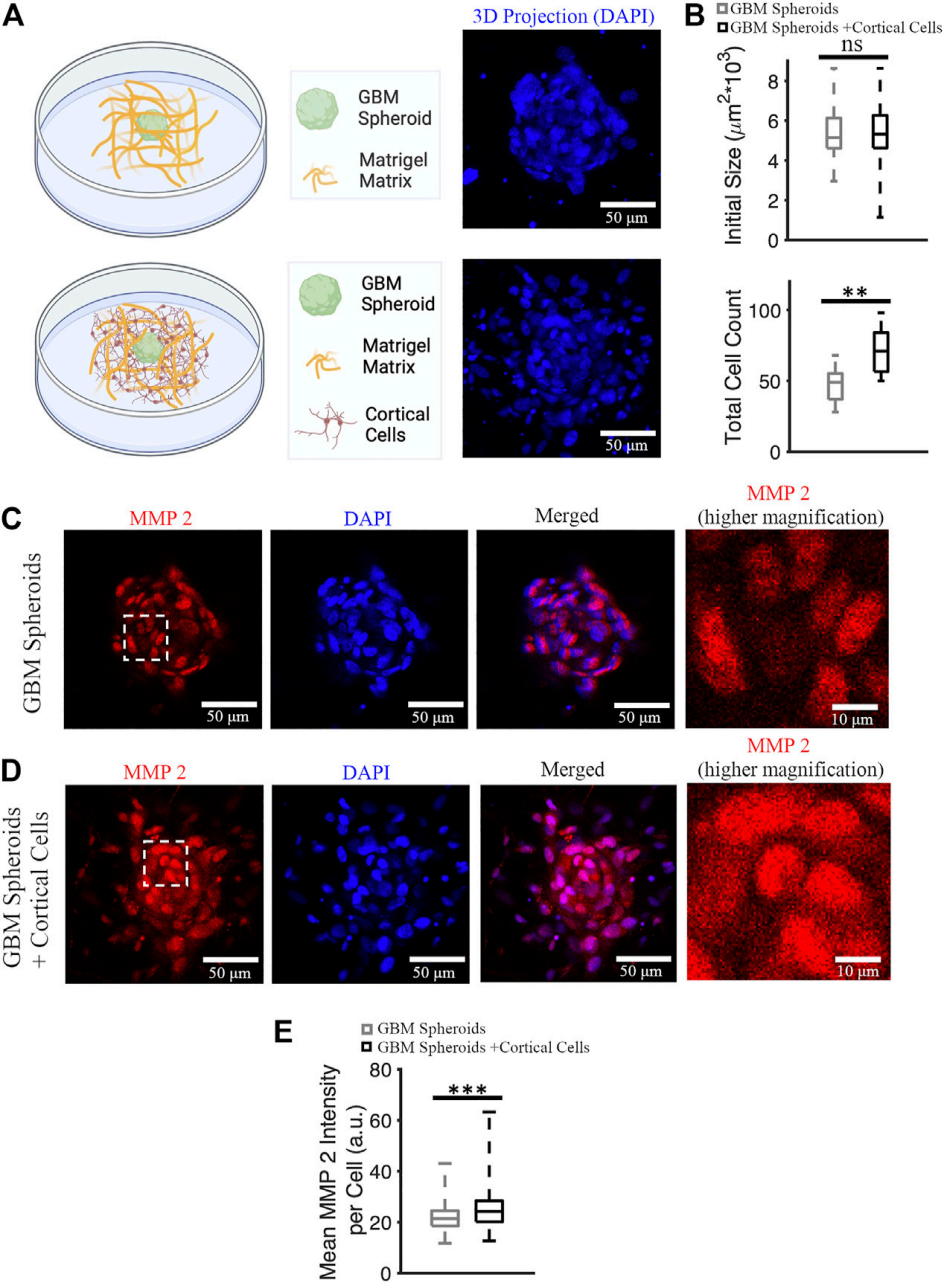
MMP 2 expression in GBM spheroids with and without cortical cells. **(A)** Schematic diagrams and 3D DAPI projections of a GBM spheroid with and without cortical cells embedded in Matrigel matrix. **(B)** Initial size and total cell count of the GBM spheroids with and without cortical cells. N = 38 and 15 GBM spheroids in each group for initial size and total cell count quantification, respectively. **(C)** MMP2 antibody staining of a GBM spheroid. The higher magnification image on the right shows the area bounded by dashed white box in higher detail. **(D)** MMP2 antibody staining of a GBM spheroid co-cultured with cortical spheroid in Matrigel. **(E)** Boxplot of the mean MMP2 intensity per cell of GBM spheroids with and without cortical cells. N = 340 cells from 34 GBM spheroids in each group. KS test was used to measure statistical significance. **p < 0.01 and ***p < 10^–8^, ns: not significant. Scale bar: 50 μm in all panels except the inset (10 μm).

GBM cells express matrix metalloproteinases (MMPs), particularly MMP 2 and 9 (Wang et al., 2003). Cancer cells remodel the extracellular matrix by secreting MMPs while they invade (Anguiano et al., 2020), and MMP 2 expression is a marker of GBM invasiveness. We stained cultures on DIV 7 (Figure 6A) with anti-MMP 2 antibody and nuclear marker DAPI, and then imaged stained cultures over the entire depth of the GBM spheroid using a confocal microscope. First, we confirmed that the initial size of the GBM spheroids (immediately after plating into Matrigel) was not significantly different between groups. Initial cross-section of GBM spheroids cultured without cortical cells was 5,379 ± 1,461 μm^2^ (*n* = 39), and initial cross-section of GBM spheroids cultured in the presence of cortical cells was 5,344 ± 1,157 μm^2^ (*n* = 38) (Figure 6B). Then, DAPI^+^ objects (cell nuclei) were counted to obtain total cell counts in the GBM spheroids. Total cell count of GBM spheroids cultured in the presence of cortical cells was significantly higher than the total cell count of GBM spheroids cultured without cortical cells (71 [56.5, 84] cells and 49 [37, 55.25] cells, respectively, KS test, *p* < 0.001) (Figure 6B). Given that the initial size of the spheroids was not significantly different, we conclude that the GBM cells proliferated faster in presence of cortical cells. To calculate mean MMP2 intensity per cell, 10 brightest cells per spheroid were considered. Expression of MMP2 was higher in GBM cells cultured in the presence of cortical cells than the GBM cells without cortical cells (24.2 [20.2, 28.4] a.u. and 21.4 [18.6, 24.5] a.u., respectively, KS test *p* < 10^–8^) (Figures 6C–E).

### 3.6 Comparison of Matrigel and matrix-free cultures

**FIGURE 7:**
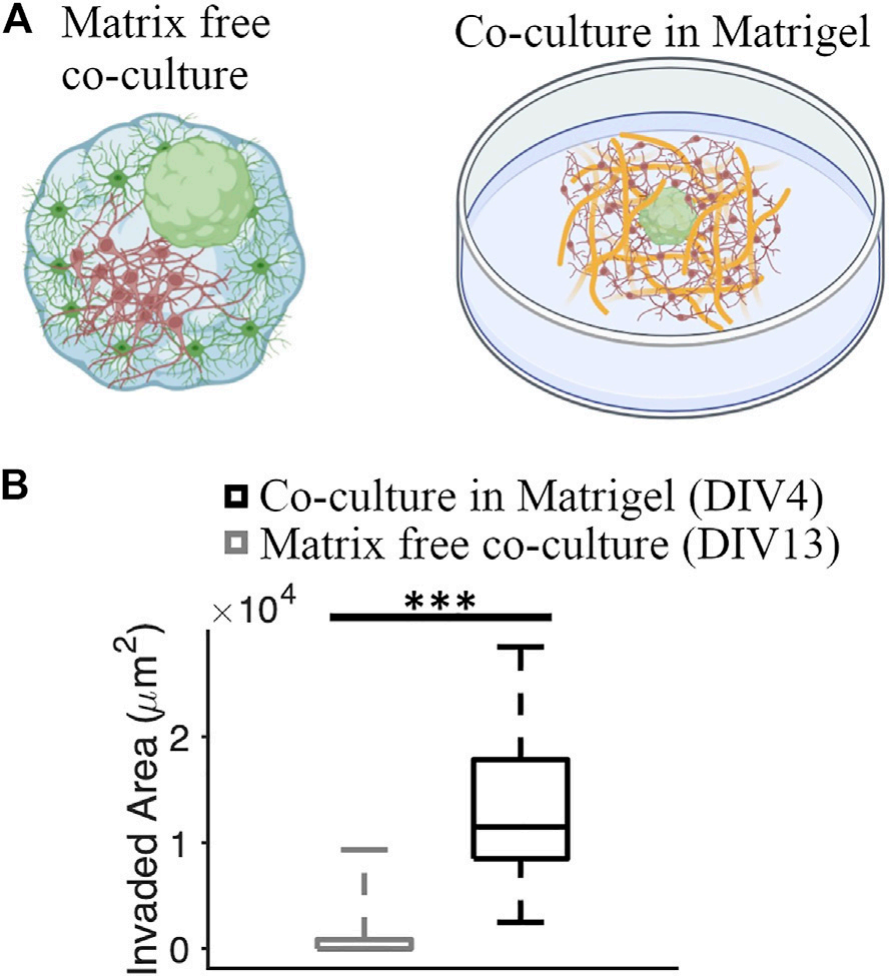
Comparison of GBM invasion between different co-culture systems. **(A)** Schematics of a matrix-free cortical-GBM spheroid co-culture and co-culture of GBM spheroid with cortical cells embedded in Matrigel matrix. **(B)** Quantification of the invaded area in different types of cultures. N = 74 and 12 GBM spheroids in co-culture with and without Matrigel matrix, respectively. KS test was used to measure statistical significance. ***p < 10^–7^.

We compared the GBM invasion between matrix-free co-culture and co-culture in the Matrigel matrix (Figure 7A). Invaded area in co-cultures embedded in the Matrigel matrix was significantly higher than the invaded area in matrix-free co-cultures (11,474 [8,493, 17,837] μm^2^ and 0 [0, 876.82] μm^2^, respectively] (KS test, *p* = 1.85 × 10^−8^) (Figure 7B).

### 3.7 GBM cells facilitate the activity of cortical neurons in Matrigel

**FIGURE 8:**
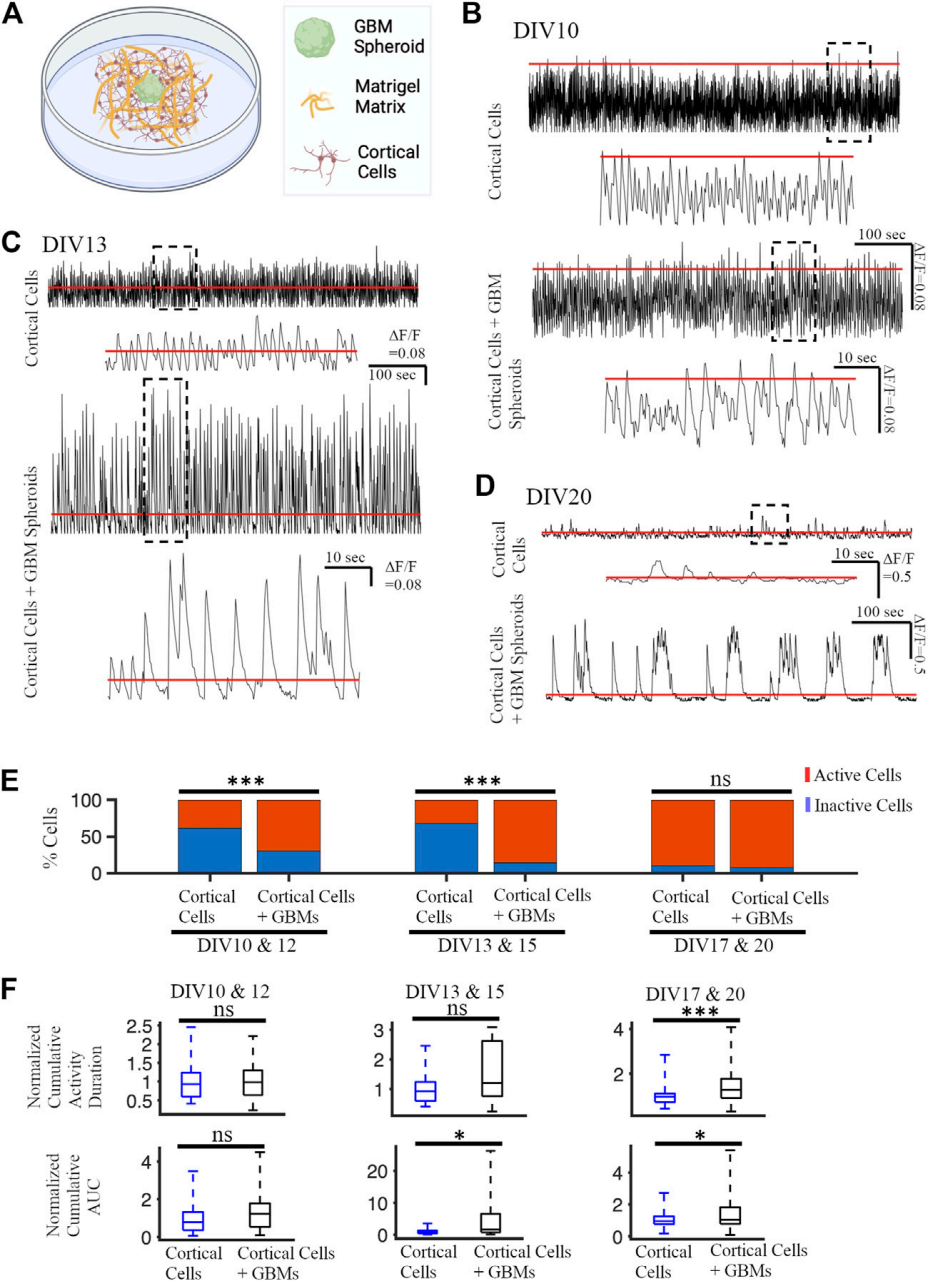
Neuronal activity in matrix co-cultures. **(A)** Schematic of a GBM spheroid with cortical cells in Matrigel. **(B)** Representative calcium traces of a cortical neuron cultured in Matrigel with and without GBM spheroids on DIV 10. The inset shows the portion of the trace contained in a dashed black box in higher detail. The red lines in all panels represent the threshold for activity analysis. **(C)** Representative calcium traces on DIV 13. **(D)** Representative calcium traces DIV 20. **(E)** Proportions of active and inactive neurons on different DIVs. N = 80 cortical neurons in each group on DIV 10 & 12 and DIV 17 & 20. N = 50 cortical cells in each group on DIV 13 & 15. The Z-score test was used to measure the statistical significance test. ***p < 0.001. **(F)** Normalized cumulative activity duration and normalized cumulative area under the curve (AUC) on different DIV. KS test was used to measure statistical significance. *p < 0.05, ***p < 10^–4^, and ns: not significant.

Recordings of neuronally-expressed jRGECO1a fluorescence changes were performed after DIV 10 (Figure 8). The activity level of neurons was different on different DIVs (Figures 8B–D). Therefore, we categorized the activity into three groups: 1) DIV 10 & 12, 2) DIV 13 & 15, and 3) DIV 17 & 20. We quantified the proportion of inactive and active cortical cells cultured with and without GBM spheroids. On earlier DIVs, the proportion of active cells in cortical cells cultured together with GBM spheroids (56 active cells out of a total of 80 cells on DIV 10 & 12 group and 43 active cells out of a total of 50 cells on DIV 13 & 15 group) was significantly higher compared to cortical cells cultured without GBM spheroids (31 active cells out of total 80 cells on DIV 10 & 12 group and 16 active cells out of total 50 cells on DIV 13 & 15 group) (Figure 8E, z-score test *p* < 0.001). On DIV 17 & 20, the proportion of active cells in cortical cells with GBM spheroids (74 active cells out of a total of 80 cells) was not significantly different compared to cortical cells without GBM spheroids (72 active cells out of a total of 80 cells) (z-score test, *p* = 0.58) (Figure 8E). We found that on early DIVs (DIV 10 & 12), normalized cumulative activity duration of neurons in co-culture with GBM spheroids was not significantly different compared to normalized cumulative activity duration of neurons without GBM spheroids (0.98 [0.64 1.3] sec and 0.93 [0.59 1.23] sec, respectively, KS test, *p* = 0.89) (Figure 8F). Also, the normalized cumulative area under the curve (AUC) above 8% ΔF/F of neurons in co-culture and without GBM spheroids was not significantly different (1.23 [0.53, 1.77] sec and 0.78 [0.36, 1.31] sec, respectively, KS test *p* = 0.1) (Figure 8F). On DIV 13 & 15, normalized cumulative activity duration of neurons in co-culture was also not significantly different compared to neurons without GBM spheroids (1.2 [0.76, 2.62] sec and 0.93 [0.59, 1.23] sec, respectively, KS test, *p* = 0.06) (Figure 8F). However, the normalized cumulative area under the curve above 8% ΔF/F of neurons in co-culture (1.57 [0.74, 6.5] sec) is significantly different compared to normalized cumulative area under the curve of neurons without GBM spheroids (0.78 [0.36 1.31] sec) (two sample KS test, *p* < 0.05) (Figure 8F). Finally, in DIV 17 & 20 group, the normalized cumulative activity duration of neurons in the presence of GBM spheroids was (1.27 [0.91, 1.77] sec) which was significantly higher than the cortical cultures without GBM spheroids (0.96 [0.72, 1.11] sec) (KS test *p* < 0.001) (Figure 8F). Also, in DIV 17 & 20 group, the normalized cumulative AUC in the presence of GBM Spheroids was (1.05 [0.78, 1.82] sec) which was significantly higher than AUC in the cortical cultures without GBM spheroids (0.94 [0.76, 1.24] sec) (KS test *p* < 0.05) (Figure 8F).

### 3.8 GBM spheroids pull cortical cells toward their invasion mantle

**FIGURE 9:**
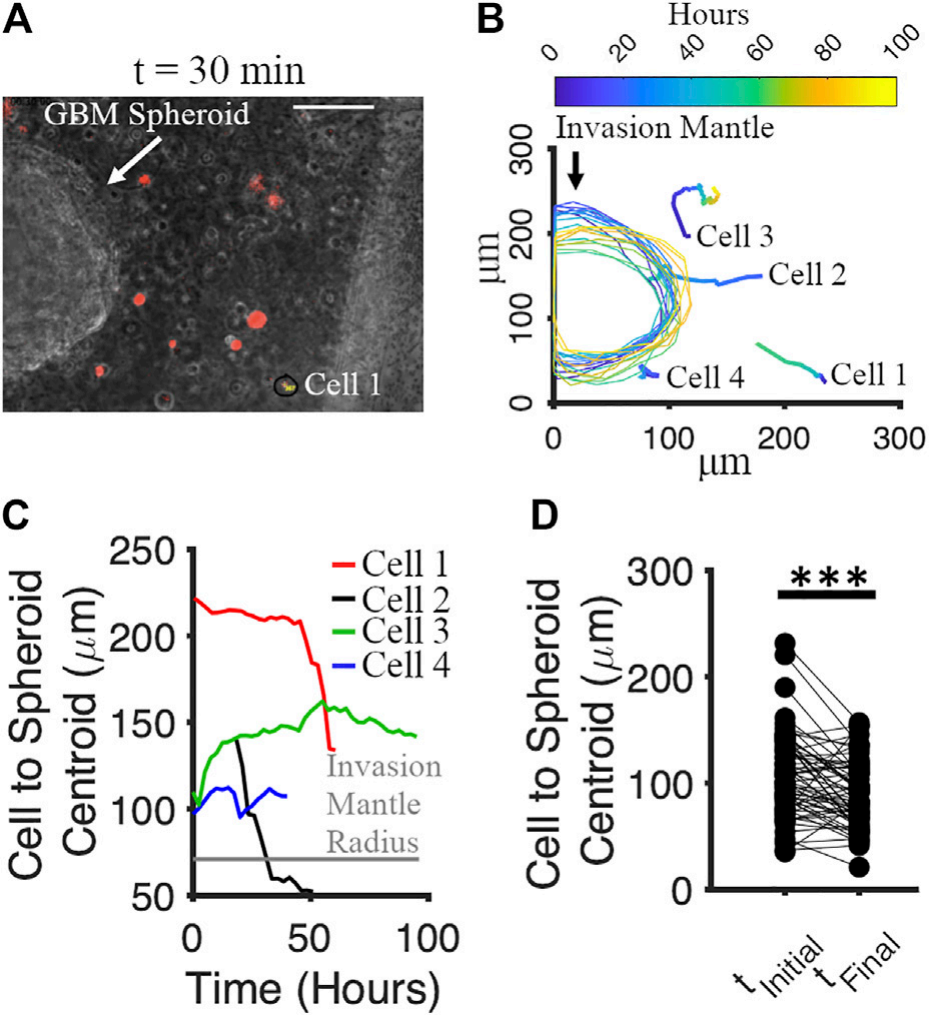
Motion of cortical cells relative to the GBM spheroid in Matrigel. **(A)** Phase micrograph of a GBM spheroid embedded in Matrigel matrix with cortical cells (labeled by Dil, red) at t = 30 min. Scale bar: 70 μm. **(B)** Tracking of cortical cells and invasion mantle of GBM spheroid over time. Colors correspond to different times after Matrigel embedding. **(C)** Distances from 4 representative cortical cells to the invasion mantle’s centroid of a GBM spheroid over time. **(D)** Distances from cortical cells to the invasion mantle’s centroid of GBM spheroid at the initial and the final time. Two-sample paired *t*-test was used to measure the statistical significance. N = 64 cortical cells. ***p < 10^–4^.

We tracked the movement of GBM spheroids and co-cultured cortical cells in Matrigel from 0 to 96 h. Cortical cells were labeled by red Dil (v22885, Invitrogen). Co-cultures were imaged every 30 min for 96 h using a time-lapse phase contrast microscope. The culture was maintained at 37°C and humidified with 5% CO_2_ during imaging. For each spheroid, 4 neighboring cortical cells and the invasion mantle were tracked with time. We analyzed the movement of 16 spheroids and 64 adjacent cells in total. One spheroid with the neighboring cells (red) is shown in Figure 9A at *t =* 30 min*.* We noticed that both spheroids’ invasion mantle and the cells’ position over time were changing (Figure 9B). We plotted the distance between the cells’ position and the centroid of the spheroid verus time (Figure 9C). Some cells were moving toward the invasion mantle, and some were moving away from it (Figure 9C). Significant decrease of the distance between cortical cells and the spheroid with time indicated that cortical cells tended to move toward the GBM spheroids (paired *t*-test: *p* = 5.58 × 10^−5^) (Figure 9D **)**.

## 4 Discussion

Our results suggest that invasiveness of patient-derived glioblastoma cells *in vitro* depends strongly on the microenvironment in which the cells are placed. GBM cells placed into a cortical spheroid exhibited limited invasiveness, with relatively short protrusions and most GBM cell soma remaining within the GBM core. In contrast, GBM cells placed into Matrigel formed long protrusions and migrated out of the GBM core. There may be multiple reasons or mechanisms that are responsible for this result. First, packing density of neurons in matrix-free 3D culture such as a cortical spheroid is as high or higher than density of neurons in cortical layers (Hasan and Berdichevsky, 2021). This packing density may impede the ability of GBM cells to migrate, and therefore, to invade. Our previous work suggested that strong neuron-to-neuron attraction and relatively weak astrocyte-astrocyte and astrocyte-neuron attraction is responsible for sorting of neurons and astrocytes into a neuron-rich core and an astrocyte-rich superficial layer in matrix-free 3D cortical cultures (Hasan and Berdichevsky, 2021). Cell-to-cell attraction may also play a role in the stability of the GBM core in cortical-GBM spheroids. Preservation of separate GBM and neuron cores suggest that GBM-GBM attraction is as strong as neuron-neuron attraction, and stronger than GBM-neuron or GBM-astrocyte attractions. A very different result emerges from embedding of GBM spheroid in Matrigel. Here, GBM cells migrate out of the spheroid core rapidly, with core ceasing to exist as a 3D object by 12 days *in vitro*. While the influence of chemical factors in Matrigel on motility of GBM cells cannot be ruled out, dissociation of the spheroid in Matrigel may be due to stronger GBM-matrix attraction compared to GBM-GBM attraction (Zaman et al., 2006). Spheroids have been described as a liquid with surface tension to explain processes such as spheroid formation and fusion (Efremov et al., 2021). Apparent tissue surface tension of spheroids may be caused by cell adhesion molecules (Foty and Steinberg, 2005) and cell contractility (Manning et al., 2010). In spheroids composed of multiple cell types, minimization of tension-related energies results in “phase separation” (Shafiee et al., 2019): a cell sorting phenomenon that we have described above for neurons, astrocytes, and GBM cells. When a spheroid is placed into an extracellular matrix such as Matrigel, competition between cell-cell and cell-matrix interactions may alter aggregation-dissociation balance. Cell migration from spheroid into the matrix may then occur. For example, surface tension of astrocytoma spheroids inversely correlated with their capacity to invade Matrigel (Winters et al., 2005). Cell-matrix interactions affecting spheroid surface tension involve matrix degradation through MMPs in addition to integrin-ECM ligand binding, and competition between tissue cohesion and affinity to ECM determined invasive potential of brain tumor spheroids in collagen (Hegedüs et al., 2006). Strong cell-matrix interactions could explain invasion of GBM cells into Matrigel we found in this work. Mechanical properties of ECM may also play a role in GBM invasiveness, but have been found to have a limited role in cell migration away from the spheroid (Nousi et al., 2020). Interestingly, when we embedded dissociated cortical cells in Matrigel, invasiveness of GBM cells increased. Robustness of the effect was confirmed by measuring it in two different GBM cell lines. The density of cortical cells in Matrigel was significantly lower than density of cortical cells in matrix-free spheroid (compare Figures 2F, 9A), and it is therefore unlikely that contact-based cell-to-cell interactions affected GBM invasiveness. We found that addition of cortical cells to Matrigel resulted in an increase in MMP2 expression in GBM core cells. This result suggests the presence of a soluble factor that is responsible for cortical-GBM cell interaction in Matrigel model, and that may be responsible for increased GBM invasiveness and proliferation. From the point of view of engineering of an *in vitro* model of GBM invasion, matrix-based model appears more preferrable as invasion is rapid and significant. Significant effect of cortical cells on gene expression and invasiveness of GBM cells suggests that these cells should be incorporated into the model to more accurately capture the microenvironment that GBM cells encounter in the brain.

Corning Matrigel is extracted from mouse sarcoma, and may include growth factors or other molecules that influence GBM invasiveness. Results in this work were obtained from 2 different batches (lots) of Matrigel, and we did not observe significant batch-related variability in GBM invasiveness or other measured parameters. Nevertheless, we designed experiments such that each run included both control and treatment groups (for example, added cortical cells). This ensured that differences due to treatment could be identified while potential influence of Matrigel batch-to-batch variability was minimized. Matrigel-based tumor invasion assay (VinciGowan et al., 2012) has been widely used for basic science and drug discovery applications. While one of the most abundant components of Matrigel, laminin (Kleinman and Martin, 2005), is present in the brain’s ECM, the other abundant component, Type IV collagen, is not typically present in the healthy brain except for lining of blood vessel walls. However, collagen, including Type IV collagen, can be produced by glioma cells, and has been associated with tumor invasion in animal models of glioblastoma (Pointer et al., 2016). Matrigel matrix may therefore represent an appropriate substrate for modeling glioblastoma invasion *in vitro*. Our Matrigel-based model, which incorporates interactions between cortical and invading glioblastoma cells, may thus be suitable for discovery of drugs that inhibit GBM invasiveness.

The lack of invasiveness in matrix-free spheroids may be due to the identity of the GBM cell line used in this work. Previously, differences in invasiveness of glioma cell line in comparison to primary glioma spheroids has been detected in corticostriatal slice cultures that have a similar neuronal packing density to our cortical spheroids (Aaberg-Jessen et al., 2013). Determination of differences of invasiveness of primary tumor spheroids versus cell lines in cortical spheroids may be a fruitful area of future investigation.

Neuronal activity in both matrix-free and Matrigel models was significantly increased due to the presence of GBM cells. As can be seen in the example traces, activity of neurons co-cultured with GBM cells self-organized into episodic paroxysmal episodes lasting longer than 1 s. We used a fluorescent calcium indicator to detect activity. Calcium levels in neurons correspond to their rate of fire (Smetters et al., 1999). Therefore, paroxysmal calcium episodes in neuronal populations can be interpreted as population-level firing bursts, or paroxysms of electrical activity with a form similar to epileptic seizures. Increase in the seizure-like activity in both of our models due to the presence of GBM cells may be modeling processes occurring in tumor-associated epilepsy. Neuronal activity in 3D cortical matrix-free spheroids may be more representative of activity in the intact cortex (Ming et al., 2020) compared to activity in dissociated neurons in Matrigel. Matrix-free co-cultures of GBM-cortical spheroids therefore represent a novel *in vitro* model of tumor-associated epilepsy. Interactions between GBM and cortical cells likely depend on soluble factors, as significant spatial separation exists between neurons and GBM cells in both models investigated in this work. We detected significant motility of cortical cells in Matrigel-based co-culture with GBM spheroid (Figure 9). This motility was absent in cultures of cortical cells in Matrigel without GBM. In co-cultures, motion of cortical cells tended to occur in the direction of the GBM spheroid. It is possible that release of factors by GBM cells activates chemotaxis of cortical cells. However, an alternative explanation could be the proteolysis of Matrigel matrix by GBM cells that results in macro-level deformation of the matrix and pulling of matrix-embedded cells toward the GBM spheroid. Mechanical stress may affect neuronal activity, potentially confounding other effects of GBM cells on neuron.

## 5 Conclusion

In this work, we established two novel *in vitro* models of glioblastoma that included primary cortical cells. We found that matrix-based model was better suited for studying glioblastoma invasion and showed increase in invasiveness due to the presence of cortical cells. On the other hand, matrix-free model was better suited for studying tumor-associated epilepsy, and showed increase in paroxysmal neuronal activity due to the presence of GBM cells. In both models, strong bidirectional interactions between GBM and cortical cells were detected.

## Data Availability

The original contributions presented in the study are included in the article/Supplementary Material, further inquiries can be directed to the corresponding author.
